# Reliability of whole mount radical prostatectomy histopathology as the ground truth for artificial intelligence assisted prostate imaging

**DOI:** 10.1007/s00428-023-03589-4

**Published:** 2023-07-06

**Authors:** Auke Jager, Arnoud W. Postema, Hans van der Linden, Peet T.G.A. Nooijen, Elise Bekers, Charlotte F. Kweldam, Gautier Daures, Wim Zwart, M. Mischi, Harrie P. Beerlage, Jorg R. Oddens

**Affiliations:** 1 Amsterdam UMC, University of Amsterdam, Department of Urology, Meibergdreef 9, Amsterdam, The Netherlands; 2 Department of Urology, Netherlands Cancer Institute – Antoni van Leeuwenhoek Hospital, Amsterdam, The Netherlands; 3 Pathology DNA, Jeroen Bosch Hospital, Henri Dunantstraat 1, 5223 GZ ’s-Hertogenbosch, The Netherlands; 4 Department of Pathology, Netherlands Cancer Institute-Antoni van Leeuwenhoek Hospital, Amsterdam, The Netherlands; 5 Department of pathology, Maasstad Ziekenhuis, Rotterdam, The Netherlands; 6 Angiogenesis Analytics, JADS Venture Campus, ’s-Hertogenbosch, AA The Netherlands; 7 Department of Electrical Engineering, Eindhoven University of Technology, Eindhoven, The Netherlands

**Keywords:** Prostate cancer, Radical prostatectomy, Ground truth, Pathology annotation

## Abstract

The development of artificial intelligence–based imaging techniques for prostate cancer (PCa) detection and diagnosis requires a reliable ground truth, which is generally based on histopathology from radical prostatectomy specimens. This study proposes a comprehensive protocol for the annotation of prostatectomy pathology slides. To evaluate the reliability of the protocol, interobserver variability was assessed between five pathologists, who annotated ten radical prostatectomy specimens consisting of 74 whole mount pathology slides. Interobserver variability was assessed for both the localization and grading of PCa. The results indicate excellent overall agreement on the localization of PCa (Gleason pattern ≥ 3) and clinically significant PCa (Gleason pattern ≥ 4), with Dice similarity coefficients (DSC) of 0.91 and 0.88, respectively. On a per-slide level, agreement for primary and secondary Gleason pattern was almost perfect and substantial, with Fleiss Kappa of .819 (95% CI .659–.980) and .726 (95% CI .573–.878), respectively. Agreement on International Society of Urological Pathology Grade Group was evaluated for the index lesions and showed agreement in 70% of cases, with a mean DSC of 0.92 for all index lesions. These findings show that a standardized protocol for prostatectomy pathology annotation provides reliable data on PCa localization and grading, with relatively high levels of interobserver agreement. More complicated tissue characterization, such as the presence of cribriform growth and intraductal carcinoma, remains a source of interobserver variability and should be treated with care when used in ground truth datasets.

**Supplementary Information:**

The online version contains supplementary material available at 10.1007/s00428-023-03589-4.

## Introduction

Artificial intelligence (AI) is gaining attention in the field of prostate cancer (PCa) imaging [1–4]. AI offers the potential to improve diagnostic accuracy and reduce operator dependency in magnetic resonance imaging (MRI), the current the standard of care in prostate imaging [4]. Additionally, AI may also play a role in advancing the clinical implementation of other imaging modalities such as multiparametric ultrasound [5].

For an AI-based imaging modality for PCa diagnosis to be effective, it must accurately localize PCa lesions and classify them into clinically relevant risk group (e.g., low, intermediate, and high-risk) [6]. The succes of any AI algorithm relies heavily on the quality of the ground truth data used in its development and training [7]. To achieve accurate data labeling, the regions assessed by the imaging modality must be categorized. For PCa diagnosis, the labeling is based on prostate histopathology, which can be obtained through prostate biopsy or radical prostatectomy specimen (RPS) [8]. While prostate biopsies are prone to underrepresenting the presence and extent of PCa, RPS provides a comprehensive view of the prostate and is considered a more suitable reference in developing AI-based imaging modalities [4].

Pathology annotation is idealy performed by an expert pathologist according to the International Society of Urological Pathology (ISUP) Grade Groups (GG) [9]. Studies have shown only fair to moderate agreement in PCa grading between pathologists, primarily involving biopsy cores instead of RPS and based on slide level agreement rather than localization of PCa [10–12]. In ISUP grading and lesion border annotation of PCa, interobserver variation can occur. Currently, there is no standardized protocol for RPS labeling as a ground truth and the reliability of such detailed pathology annotation is not well understood.

In a multicenter trial aimed at developing an AI-based image analysis algorithm for PCa diagnosis on three-dimensional multiparametric transrectal prostate ultrasound, a comprehensive model was developed to provide a ground truth based on RPS [13]. In the current paper, we describe the development of a standardized protocol for RPS annotation (part 1) and the results of a study evaluating the feasibility and reliability of this protocol (part 2).

## Methods

### Part I: creating the annotation protocol

The whole-mount RPS pathology protocol was developed by an expert panel consisting of urologists and urology residents (AJ, AP, JO, HB), uropathologists (HL, PN, AH, CW, KK), and engineers (MM, WZ). Its purpose is to provide a reliable ground truth for correlation of pathology (location and grading) with prostate imaging.

The expert panel conducted three consensus meetings to refine and finalize the protocol. The first version was tested on two RPS, each annotated by five uropathologists (PN, AH, CW, KK, EB) and evaluated in a second consensus meeting. After this meeting, the second version of the protocol was developed and applied on four RPS, each annotated by two uropathologists. In the third consensus meeting, the definitive version of the annotation protocol was determined.

The full version of the standard operating procedure of the annotation protocol is provided as supplementary materials 1. Identification of Gleason patterns (GPs) and secondary tumor characteristics in the current protocol was performed according to the growth patterns defined in the ISUP guidelines 2019 [9].

### Stepwise summarized annotation protocol

#### Annotation of prostate cancer

##### Gleason patterns

Clinical evaluation according to the ISUP Grade Groups often combines areas that contain different GPs. However, when training an AI algorithm for PCa diagnosis on imaging, it is crucial that the algorithm recognizes specific image characteristics that are distinct for different GPs and are a result of tissue morphology. To achieve this, the expert panel decided that it should be avoided to annotate areas that contain a mixture of GPs (such as Gleason Score 3+4=7). Instead, it was decided to annotate cancerous tissue areas that contain solely GPs 3, 4, or 5, when possible. The pilot study showed some areas containing both GP 3 and 4 or 4 and 5 are too heterogeneous to separately annotate. For these areas, the option to annotate areas as Gleason Scores was incorporated in version 2 of the protocol.

##### Secondary tumor characteristics

Cribriform growth (CG) and/or intraductal carcinoma (IDC) are clinically important prognostic factors in PCa and are therefore separately annotated [9, 14, 15].

#### Annotation of benign abnormalities

Prostatitis and high-grade prostatic intraepithelial neoplasia are benign conditions that can sometimes lead to false positive results in prostate imaging. In order to understand the impact of these conditions, it is important to determine their presence in a given prostate.

##### Level of precision

Due to medical imaging resolution and inaccuracies when correlating imaging to pathology, annotation precision below 0.5 mm was deemed unnecessary for the purpose of the current protocol. Evaluation of the pilot study showed a wide variation in the level of precision between pathologists, causing a variation in time expenditure due to unnecessarily precise annotations. To ensure uniformity in the level of precision between pathologists, the scale at which cancerous tissue is annotated was set at 1 or 2 mm. Additionally, the polygon line thickness in the annotation tool was standardized at 0.2mm, which prevents overly detailed annotations.

### Part II: feasibility and reliability of the final annotation protocol

Part II of this study was performed using the definitive version of the annotation protocol on full-mount RP slides originating from ten patients prospectively included in August and September 2021 at the Amsterdam University Medical Centres and the Netherlands Cancer Institute. These patients participated in a multicenter trial currently being carried out in the Netherlands (NCT04605276) [13]. Creating the annotation protocol was part of this trial and the protocol does not interfere with regular clinical evaluation. The study was approved by the institutional review board, reference number 2020_268#B202178. The ten prostates were randomly assigned to five uropathologist. Each pathologist annotated four prostates; each prostate was annotated by two different pathologists. The participating pathologists had at least 7 years of experience with prostate pathology and were trained at different centers in the Netherlands. Pathologists were blinded to each other and for clinical patient characteristics, including MRI and biopsy results.

#### Whole-mount histopathology slide preparation

The prostate specimen was fixated in formalin for at least 24 h. After, fixation specimens were sectioned from apex to base in 4-mm slices using a TruSlice specimen cut-up system (Cellpath Ltd, Newtown, UK). The prostate slices were fitted in cassettes, embedded in paraffin, and cut into whole-mount pathology slides (4 μm thick).

Whole-mount pathology slides were scanned on high resolution (40× enlargement, 20× objective, 2.1 camera lens) using a Pannoramic 1000 Digital Slide Scanner (3DHISTECH, H-1141 Budapest, Öv u. 3., Hungary) and uploaded to a web-based pathology annotation tool (Slidescore, Amsterdam, the Netherlands). The parasagitally cut apical and basal pathology slides were not included for annotation in the current study.

#### Study outcomes

**Fig. 1:**
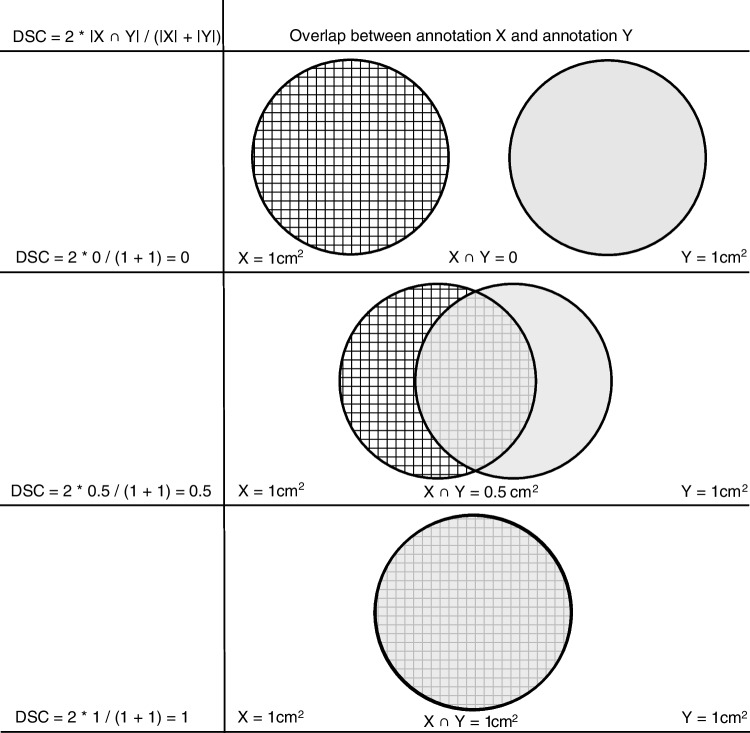
Examples for DSC calculation. DSC, Dice similarity coefficient

The primary outcome for this study was to evaluate the accuracy of PCa tissue localization and grading. This was evaluated by analyzing the surface-based interobserver agreement per RPS between pathologists expressed as the weighted dice similarity coefficient (DSC). DSC is defined as: 2 × |*X* ∩ *Y*| / (|*X*| + |*Y*|). Weighted DSC was defined as $\frac{\left(X+Y\right) DSC}{2Z}$. *X* and *Y* are the surface areas annotated by pathologists 1 and 2 on a single pathology slide. *X* ∩ *Y* is the area where *X* and *Y* overlap. *Z* is the mean surface area annotated by pathologists 1 and 2 on all pathology slides belonging to one RPS. The DSC is a value that ranges from 0 to 1, where 0 indicates no overlap between two annotated surface areas and 1 indicates perfect overlap (Fig. 1) [16]. The weighted DSC per RPS is the sum of the weighted DSC from each slide. Weighted DSC was calculated for PCa, defined as any GP 3 or higher, for clinically significant PCa (csPCa), defined as any GP 4 or higher, and for CG and/or IDC.

The secondary outcomes were the level of agreement in tissue characterization on a per-slide level and agreement on localization and grading of the index lesion. The agreement in tissue characterization on a per-slide level was expressed as Fleiss kappa (interobserver variability). Kappa values were interpreted as follows: Poor agreement for kappa <0.00, slight agreement for kappa is 0.00 to 0.20, fair agreement for kappa is 0.21 to 0.40, moderate agreement for kappa is 0.41 to 0.60, substantial agreement for kappa is 0.61 to 0.80, and almost perfect agreement for 0.81 to 1.00. Agreement on a slide level was evaluated for [1] any PCa, [2] csPCa, [3] primary and secondary GP, [4] presence of CG/IDC, [5] presence of a minor pattern 5. Any PCa was defined as any GP 3 or higher, csPCa as any GP 4 or higher. Primary, secondary, and minor GPs were defined according to the 2019 ISUP consensus meeting [9]. Primary GP was defined as the pattern with the largest surface area. Secondary GP was defined as the pattern with the second largest surface area, or, if there was a higher GP present, as the highest GP (provided that the surface area accounts for ≥5% of the total tumor area). A minor pattern 5 was defined as a GP 5 that accounts <5% of the total tumor area in a slide.

The index lesion was defined as the lesion with the highest ISUP GG with a surface area of ≥0.5cm^2^. If multiple lesions with the same ISUP GG are annotated within one RPS, the lesion with the highest volume was considered to be the index lesion. To properly compare grading of the index lesions, the Gleason patterns were translated to ISUP GG according to the definitions provided by the ISUP guidelines [9]. The agreement on grading and localization was expressed as a percentage and DSC, respectively.

For both the primary and secondary outcomes, the results of the five participating pathologists were bundled to allow for comparison between two observers (pathologists 1 and 2).

Additional evaluation included time expenditure of executing the protocol per annotated prostate. Time expenditure was reported by the pathologist performing the annotations.

## Results

A total of 10 RPS consisting of 74 whole mount pathology slides were used to evaluate the reliability of the definitive version of the protocol.

### Lesion level

**Fig. 2:**
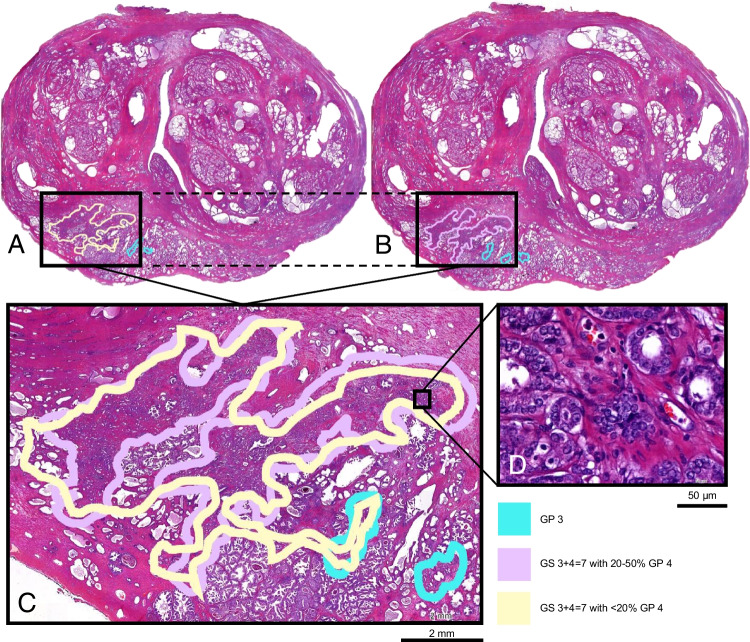
Pathology slide from the RPS with the lowest DSC. **A** Surface area of PCa = 0.30 cm^2^ and csPCa = 0.26 cm^2^. **B** Surface area of PCa = 0.30 cm^2^ and csPCa = 0.28 cm^2^. The lower DSC can be attributed to differences in annotation style. P1 (A) annotated larger area as GS 3+4=7 with <20% GP 4, where P2 (B) chose to annotate a similar area as two separate areas of GS 3+4=7 with 20–50% GP 4 and 1 area of GP 3. **C** Overlay of the annotations from P1 (A) and P2 (B), differences in annotated areas are minimal; however, DSC is relatively low at 0.74 for PCa and 0.76 for csPCa. Scale bare 2 mm. **D** Enlarged area contains scale bar 50 μm. RPS, radical prostatectomy specimen; DSC, dice similarity coefficient; PCa, prostate cancer; csPCa, clinically significant prostate cancer; P1, pathologist 1; P2, pathologist 2; GS, Gleason score; GP, Gleason pattern

**Fig. 3:**
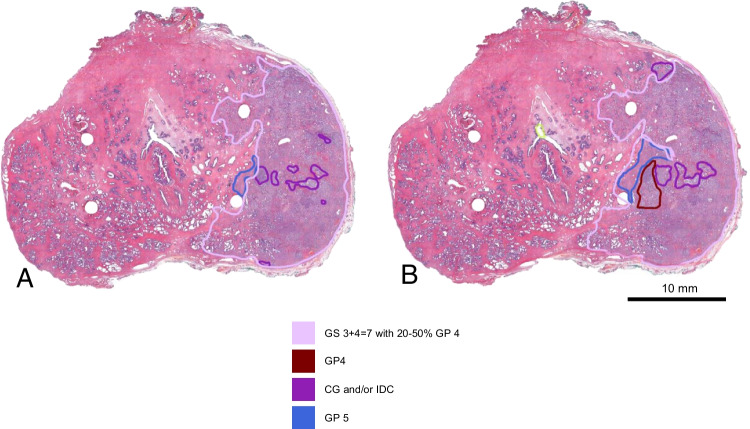
Slide from the prostate with almost perfect agreement on localization and characterization. DSC for csPCa = 0.96. **A** P1, surface areas: csPCa = 2.02 cm^2^, CG/IDC = 0.09 cm^2^, Gleason 5 = 0.04 cm^2^, Gleason 4 = 0 cm^2^. **B** P2, surface areas: csPCa = 2.14 cm^2^, CG/IDC = 0.12 cm^2^, GP 5 = 0.10 cm^2^, GP 4 = 0.08 cm^2^. Both pathologist agree on the presence of GP 5 and CG/IDC; however, the extent and localization differ, leading to DSCs of 0.56 for gleason 5 and 0.47 for CG/IDC. Scale bar 10mm. DSC, Dice similarity coefficient; P1, pathologist 1; P2, pathologist 2; csPCa, clinically significant prostate cancer; CG, cribriform growth; IDC, intraductal carcinoma; GP, Gleason pattern; GS, Gleason score

The average total surface area annotated by the pathologist in all ten RPS was 34.55 cm^2^ for PCa and 31.80 cm^2^ for csPCa. Overall agreement on localization, expressed as weighted DSC, was 0.91 for any PCa and 0.90 for csPCa. Agreement varied between prostates, with a tendency towards a lower DSC with smaller areas of PCa (Table 1). CG/IDC was annotated in four out of ten prostates and showed an overall weighted DSC of 0.64 cm^2^ (Table 2). Figures 2 and 3 show the worst and best performing pathology slides.

**Table 1:** Total surface area annotated for PCa and csPCa by the pathologists for each prostate in square centimeters. Agreement on localization is given as weighted DSC. *PCa* prostate cancer, *csPCa* clinically significant prostate cancer, *DSC* Dice similarity coefficient, *P1* pathologist 1, *P2* pathologist 2

Prostate	RPS surface area (cm^2^)	Annotated PCa (cm^2^)		Weighted DSC	Annotated csPCa (cm^2^)		Weighted DSC
		P1	P2		P1	P2	
1	95.0	0.30	0.31	0.74	0.26	0.28	0.76
2	93,9	0.92	0.77	0,84	0.70	0.77	0.81
3	94.6	1.31	1.30	0.94	1.29	1.28	0.94
4	46.7	2.05	2.05	0.87	2.05	1.77	0.90
5	69.1	2.83	3.44	0.87	2.82	3.44	0.87
6	144.7	3.49	3.83	0.91	3.05	3.68	0.89
7	76.9	3.88	3.25	0.87	3.71	3.04	0.86
8	22.5	4.44	4.29	0.82	4.21	3.95	0.82
9	22.6	5.19	5.73	0.94	5.17	5.73	0.94
10	19.9	9.73	9.60	0.96	8.96	7.08	0.85
Total	686.01	34.14	34.57	0.91	32.22	31.02	0.90

**Table 2:** Total surface area annotated for CG/IDC by the pathologists for each prostate in square centimeters. Agreement on localization is given as weighted DSC. The poor agreement for prostate 6 is also visualized in Fig. 4. *CG* cribriform growth, *IDC* intraductal carcinoma, *DSC* dice similarity coefficient, *P1* pathologist 1, *P2* pathologist 2

Prostate	Annotated CG/IDC (cm^2^)		
	P1	P2	Weighted DSC
6	0.91	0.012	0.01
7	0.21	0.16	0.63
9	0.09	0.16	0.39
10	1.87	2.28	0.79
Total	3.08	2.61	0.64

### Slide level

A total of 74 whole mount pathology slides, originating from ten RPS, were annotated using the definitive version of the pathology protocol. Agreement on the presence of any PCa was perfect. Pathologists were in 100% agreement that 48 out of 74 pathology slides contained PCa (GP ≥ 3). For csPCa, agreement was almost perfect with a Fleiss kappa of .915 (95% confidence interval (CI) .689–1.0). Agreement on primary and secondary GP was almost perfect and substantial, with Kappa of .819 (95% CI .659–.980) and .726 (95% CI .573–.878), respectively. Table 3 gives an overview of interobserver variability for each annotation category.

**Table 3:** Interobserver variability for different annotation categories on a per pathology slide level

Category	#P1*	#P2*	Fleiss kappa (95% CI)	*P*-value
All slides	74	74	–	–
Any PCa	48	48	1.0 (.774–1.0)	.000
csPCa	45	48	.915 (.689–1.0)	.000
Primary GP	NA	NA	.819 (.659–.980)	.000
Secondary GP	NA	NA	.726 (.573–.878)	.000
GP 3	43	43	.727 (.501–.954)	<.001
GP 4	45	48	.915 (.689–1.0)	.000
GP 5	1	2	.000 (.000–.206)	.860
Minor pattern 5	7	3	.357 (.131–.583)	.002
CG/IDC	13	16	.701 (.474–.927)	<.001
Prostatitis	17	21	.507 (.280–.733)	<.001

*Number of slides in which the category was annotated by P1 or P2

*PCa* prostate cancer, *csPCa* clinically significant prostate cancer, *GP* Gleason pattern, *CG* cribriform growth, *IDC* intraductal carcinoma, *P1* pathologist 1, *P2* pathologist 2

### Index lesion

The weighted overall DSC for the index lesions of all ten RPS was 0.92. The mean DSC was 0.89. Agreement on ISUP GG was seen in 70% of the index lesions (Table 4).

**Table 4:** Index lesion characteristics and agreement between P1 and P2. *P1* pathologist 1, *P2* pathologist 2, *ISUP GG* International Society of Urological Pathology Grade Group, *DSC* Dice similarity coefficient

RPS	Index lesion surface area (cm^2^)		ISUP GG		Weighted DSC
	P1	P2	P1	P2	
1	0.26	0.28	2	2	0.76
2	0.35	0.32	4	3	0.90
3	1.23	1.20	3	3	0.95
4	2.05	1.77	3	3	0.90
5	2.80	3.03	2	3	0.90
6	3.07	3.31	3	3	0.94
7	2.85	2.26	2	2	0.84
8	1.81	1.84	3	5	0.80
9	5,18	5,78	2	2	0.94
10	9.63	9.66	3	3	0.97
Total	29.45	29.23	–	–	0.92

### Time intensity

The median annotation time per prostate for the first version of the protocol was on average 3 h (range 1–5). For the definitive version of the protocol, average annotation time decreased to 2 h (range 1–4).

## Discussion

There is a need for more efficient and reliable imaging for PCa diagnosis. Although MRI has shown to significantly improve patient selection prior to biopsy, its limited availability, high costs, and substantial interobserver variability remain an issue [17]. With the 2022 European Union recommendations to include PCa in population-based screening programs, the demand for accurate and reliable imaging will only intensify. AI-assisted automated detection methods for PCa have the potential to address these issues [13]. However, to effectively train and validate these diagnostic methods, it is crucial to assess the reliability of the ground truth. In this particular case, the ground truth is represented by RPS histopathology, which serves as the reference standard for PCa diagnosis [18, 19]. Studies that utilize prostate histopathology as the reference standard often fail to evaluate the reliability of their reference standard [20, 21]. In cases where evaluations are conducted, they typically focus on the accuracy of the correlation between pathology and imaging, overlooking the assessment of pathology annotation itself. This often involves relying on a single pathologist to annotate pathology slides, despite the well-known interobserver variability in PCa grading [11, 12, 22]. Furthermore, existing studies mainly report on grading agreement at the slide level, leaving a gap in our understanding of the agreement on the localization of PCa lesions among pathologists.

The current study aimed to address this gap in knowledge and demonstrated outstanding agreement in the localization of PCa, csPCa, and the index lesion, with weighted DSCs of 0.91, 0.90, and 0.92, respectively. Moreover, agreement on presence of PCa and csPCa on a per-slide level was near-perfect. These results demonstrate that the proposed protocol provides a reliable reference or ground truth for PCa localization and characterization.

**Fig. 4:**
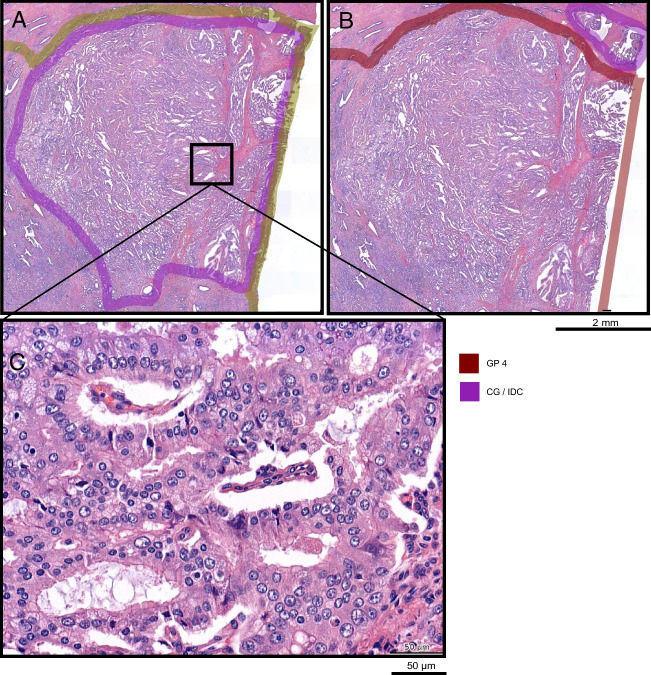
Difference in characterization of a tumor area. Agreement on localization and presence of PCa is excellent (weighted DSC = 0.94). There is no agreement on characterization the CG/IDC. **A** P1 annotated a substantial area as CG/IDC (0.25cm^2^). **B** P2 annotates the same area as GP 4, without CG/IDC. Scale bar 2mm. **C** Area enlarged. Scale bar 50 μm. Pca, prostate cancer; DSC, Dice simillarity coefficient; CG/IDC, cribriform growth/intraductal carcinoma; GP, Gleason pattern

The characterization of secondary tumor characteristics (CG/IDC) proved more challenging. On a per-slide level, agreement was substantial; however, there was less agreement on localization with an overall weighted DSC of 0.64. This can be partly attributed to a difference in annotation precision; some pathologists annotate many small areas of CG/IDC where others annotate fewer but larger areas. However, it also reflects a discrepancy in the interpretation of what should be classified as CG/IDC. The limited agreement on CG/IDC is a known issue [23]. Van der Slot et al. showed only moderate agreement between five pathologists on a per prostate level in 80 RPS [10]. While the current study demonstrated a modest improvement in agreement on a per-slide level, it also shows that the characterization of various types of GP 4 remains a complex task [22]. Figure 4 illustrates a case that exemplifies the difficulties in characterizing CG/IDC. In this case, the pathologists involved did not come to a consensus on the presence of CG/IDC in a larger area, even after revisiting the case. A third pathologist found that the pattern was not entirely consistent with the typical CG/IDC pattern. Instead, it was considered to be a borderline case, described in previous literature as “complex fused” [22].

On a per-slide level, agreement of primary and secondary GP was substantial to almost perfect. For clinical grading of PCa according to ISUP, an often-voiced concern is the interobserver variability between pathologists, reaching fair to moderate agreement for Gleason grading [11, 24]. A possible explanation of the relatively high agreement in the current study is that the detailed annotation protocol resulted in more careful evaluation of the pathology slides.

**Fig. 5:**
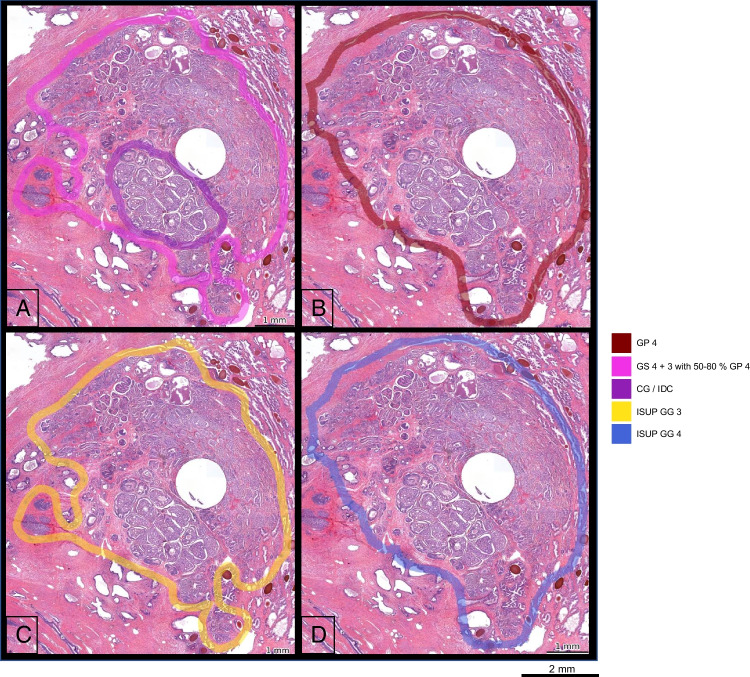
Discordance in grading between pathologists. **A** P1 annotated this area as GS 4+3 with 50–80% GP 4 and an area of GP 4 with CG/IDC, **B** P2 annotated the entire area as GP 4. **C** Translation of A into ISUP GG 3. **D** Translation of B into ISUP GG 4. The similarities between A and B are high (a lesion with a high percentage of GP 4); the difference seems larger after translation to ISUP GGs. Scale bar 2 mm. P1, pathologist 1; P2, pathologist 2; GS, Gleason score; GP, Gleason pattern; CG, cribriform growth; IDC, intraductal carcinoma; ISUP, International Society of Urological Pathology; GG, grade group

As the index lesion holds the most clinical relevance, a focused analysis was conducted to evaluate the localization and grading of the lesion [25]. The process of translating adjacent areas that were previously annotated as separate GPs into ISUP GGs, as depicted in Fig. 5, yielded a 70% concordance rate in terms of the ISUP GG assigned to the index lesion. This conversion approach also facilitates comparisons between different annotation protocols and clinical practice. The three cases of disconcordance between pathologists show the benefit of the protocol used in the current study. A discordance in ISUP GG can imply a substantially different interpretation on tissue morphology; however, examining the original annotations according to the study protocol shows that discrepancies in tissue characterization are often minor (Fig. 5).

This study has several shortcomings. Although the surface-based agreement provides many data points, the number of occurrences for some tissue types was limited (e.g., GP 5) and no reliable analysis of agreement on these tissue types could be performed. Furthermore, due to the design of the protocol, no surface-based analysis on the grading of separate GPs could be performed. To obtain a more comprehensive understanding of the agreement among pathologists for different grade classifications, further extensive analysis involving a larger sample size and annotations by additional pathologists will be necessary. However, grading of the index lesion showed an excellent surface based agreement as well as agreement on ISUP GG in seven out of ten RPS. The pathologists who participated in this study possessed extensive experience in prostate pathology. They underwent training and worked in different centers within the same country. While their expertise and diverse backgrounds contribute to the robustness of the study, it is important to acknowledge that the results may have limited generalizability to an international setting or in a setting with less experienced pathologists. Lastly, the time intensity of the study protocol was substantial. Adjustments made in the final protocol did decrease annotation time, but it remained time-intensive at an average of 2 h per prostate.

## Conclusion

The results of this study indicate that the RPS pathology can be utilized for training and developing AI-based imaging modalities. Through standardization and evaluation of annotation methods, the current study achieved relatively a high level of agreement between experienced pathologist, with substantial to almost perfect agreement for PCa localization and grading. Agreement on the presence of more complex tissue morphology (e.g., CG/IDC) remains limited, and their inclusion in a ground truth dataset should be approached with caution.

## Supplementary information

ESM 1 (PDF 980 kb)

## Data Availability

The datasets used and/or analyzed during the current study are available from the corresponding author on reasonable request.
