# Supplementary material for: Reliability of whole mount radical prostatectomy histopathology as the ground truth for artificial intelligence assisted prostate imaging
Source: Virchows Arch. 2023 Jul 6;483(2):197–206. doi: 10.1007/s00428-023-03589-4 (PMC10412486; doi:10.1007/s00428-023-03589-4)

Prostate Pathology Annotation Protocol

3D MULTIPARAMETRIC ULTRASOUND FOR PROSTATECANCER DETECTION (3D mpUS for PCa)

Version	3.0
Date	04-02-2022
Authors	Auke Jager<br>Hans van der Linden<br>Peet Nooijen

Table of Contents

Background 2

.... 4

Starting an annotation 4

General 5

1. Annotation of areas containing tumor 5

2. Prostatitis & HG-PIN 6

3. Submitting a patient 6

Notes 7

What to do when in doubt? 7

Areas of mixed tissue types (Gleason Patterns 3 & 4 or 4 & 5) 7

Level of detail 8

Cribriform growth vs. Intraductal carcinoma 8

Background

This pathology annotation protocol is part of the multicenter clinical trial: Three-dimensional Multiparametric Ultrasound for Prostate Cancer Detection (3D mpUS for PCa). The goal of this study is to train a machine learning-based algorithm that can detect prostate cancer on multiparametric transrectal ultrasound. To train this algorithm, a reliable ground truth is key. The ground truth will consist of full mount prostatectomy histopathology. The algorithm will need to be able to recognize, characterize and localize prostate cancer. Therefore, the ground truth pathology data will have to be more detailed than that provided by regular clinical evaluation of prostatectomy specimen. Different Gleason patterns will need to be separately annotated, so that the algorithm learns to differentiate between the morphologically different types of prostate cancer. Furthermore, high-grade prostatic intraepithelial neoplasia and prostatitis will have to be separately annotated because they can cause false-positives on imaging.

Summarized annotation protocol

1. Annotate areas containing tumor using the polygon on a scale of 1 or 2 mm
2. Annotate areas containing HG-PIN and prostatitis on a scale of 1 or 2 mm
3. Check the “finished with slide”-box when all steps for a slide are finished
4. Click ‘’submit’’ to save your progress

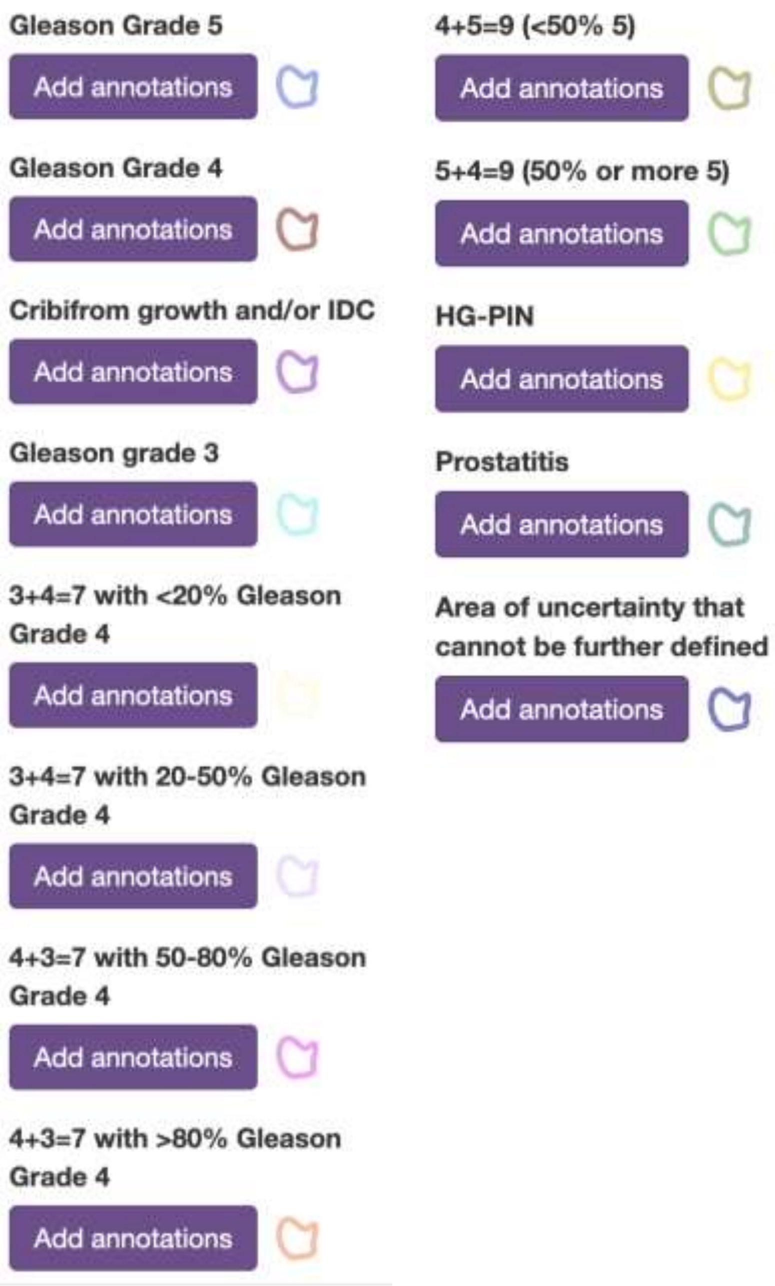

Starting an annotation

General

On the left of the screen, you can select an annotation option (e.g. Gleason Grade 5 etc.) To produce an annotation, select the correct tissue type and click ''add annotation''. Click **polygon** to start annotating

1. Annotation of areas containing tumor

- The polygon always needs to be closed to finish the annotation
- To reduce the variability in precision between pathologist, always annotate areas on a scale of either 1 or 2 mm. The scale is visible in the right down corner of the screen;
- Areas containing prostate cancer need to be annotated with a precision of 0.5mm. Areas smaller than 0.5mm do not need to be separately annotated. The scale can be used as a reference;
- The polygon line always has a thickness of **0.2mm**, this can also be used as a reference;

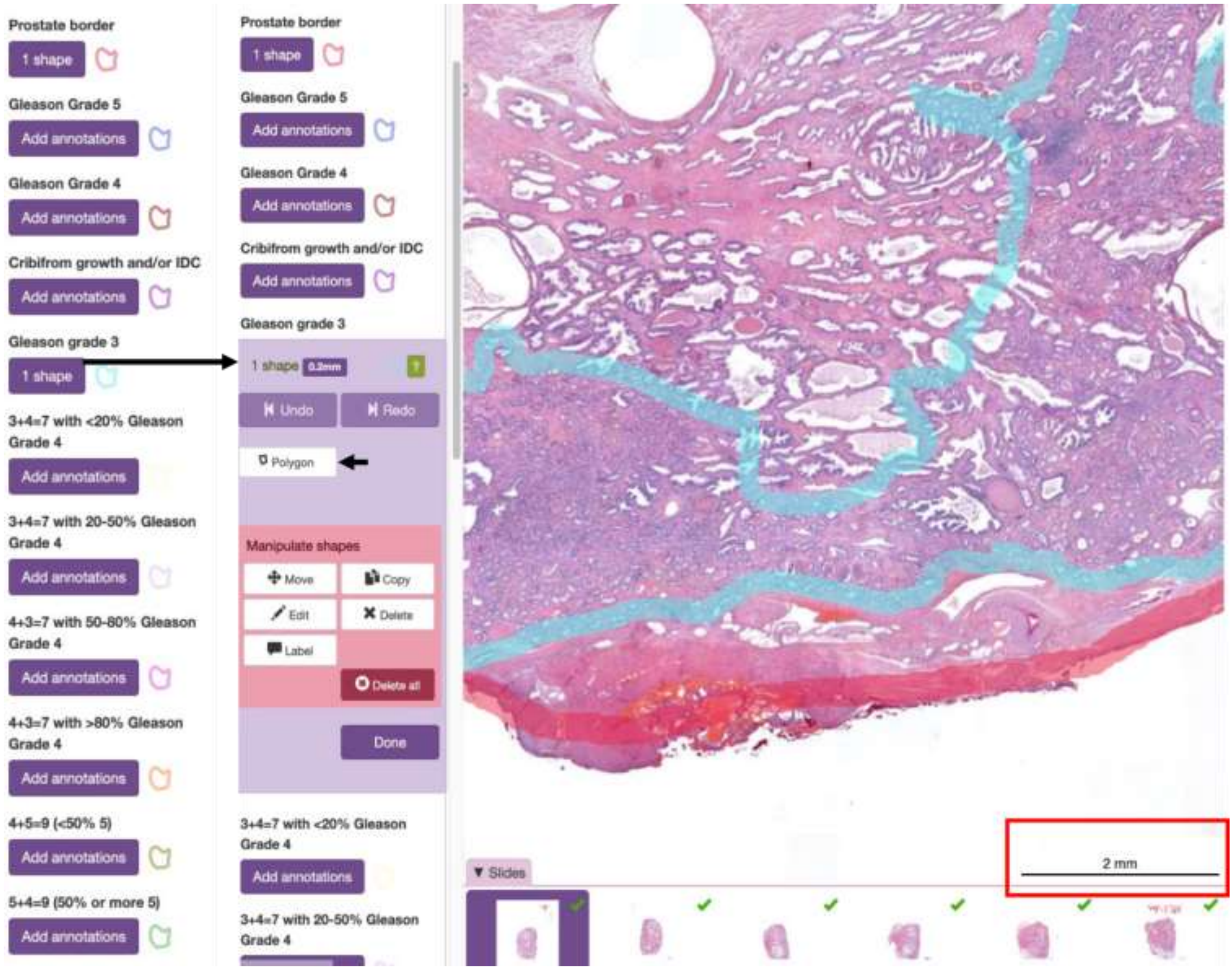

2. Prostatitis & HG-PIN

The pilot study showed that annotating prostatitis with a high level of precision is time intensive. It was also deemed unnecessary for training the algorithm. However, it still is important to know if there is an area that contains prostatitis, to evaluate possible false positive results. Roughly delineating these areas is sufficient. This also applies to areas of HG-PIN. The slide below gives an example of how to delineate prostatitis.

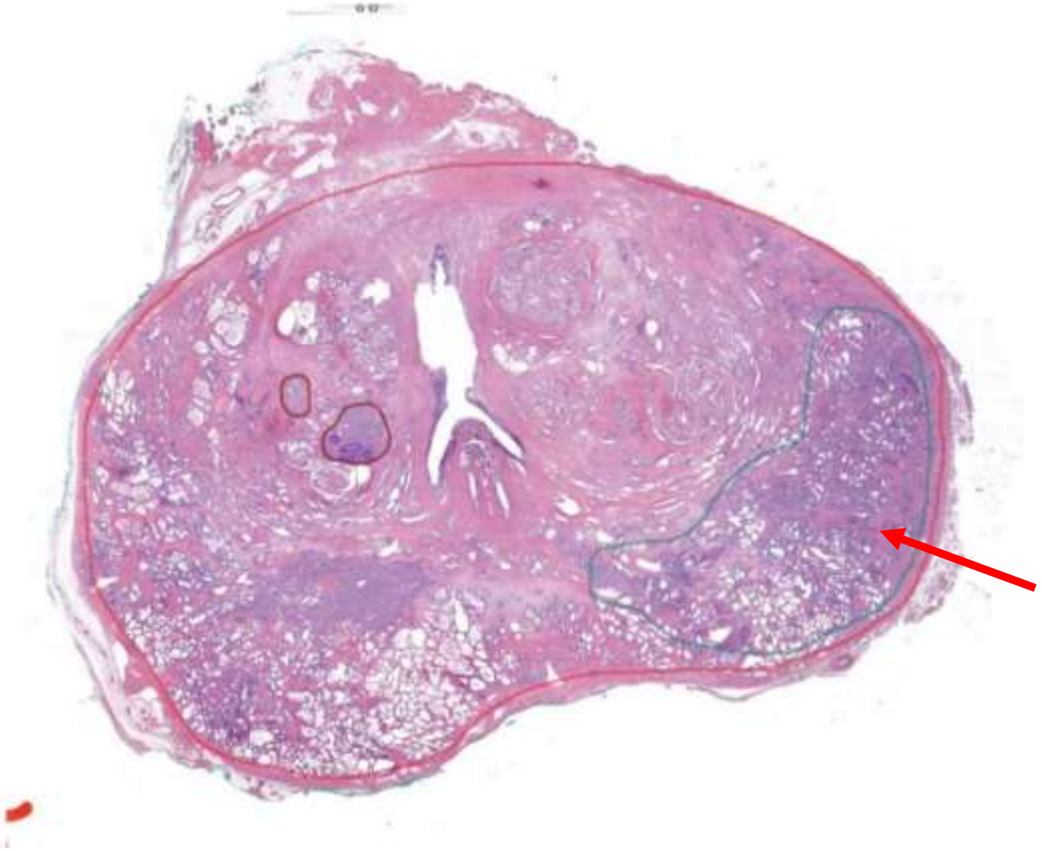

3. Submitting a patient

When all steps of the protocol have been competed, check the **''finished with slide''** box and click **''submit''**. To save your progress before finished the prostate you can alsof click **''submit''.**

Notes

What to do when in doubt?

When in doubt on the characterization of a specific area, the following steps are to be applied:

1. Annotate the area according to what you think fits best (e.g. Gleason Pattern 4);
2. Click on **''label''** under **''manipulate shapes''**;
3. Click on the area of doubt;
4. A window pops up that says ''**add a label to shape''**;
5. Insert a comment explaining what you are doubting on, select ''**always''** under **''when to show label''** and click **''confirm''**;
6. The label will appear over the annotation in question and a second pathologist will review the area.

Areas of mixed tissue types (Gleason Patterns 3 & 4 or 4 & 5)

The pilot study has proven that some areas are to heterogenous to separately delineate different. However, to train the algorithm to differentiate between different levels of aggressivity of prostate canncer, it is important to separately train on areas with Gleason pattern 3, 4 and 5. For this reason 6 different annotation options have been added to the protocol, based on the percentage in which different Gleason patterns are presen:

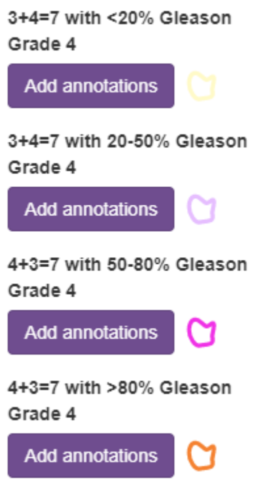

1. Mixed tissue: 3 & 4 with <20% Gleason pattern 4
2. Mixed tissue: 3 & 4 with ≥20% and <50% Gleason pattern 4
3. Mixed tissue: 4 & 3 with ≥50% and <80% Gleason pattern 4
4. Mixed tissue: 4 & 3 with ≥80% Gleason pattern 4
5. Mixed tissue: 4 & 5 with <50% Gleason pattern 5
6. Mixed tissue: 5 & 4 with ≥50% Gleason pattern 5

<u>Important:</u>

- Preferably Gleason patterns are annotated separately;
- Cribriforme growth or IDC need to be separately annotated within areas of mixed tissue.

Level of detail

The pilot study has shown that the level of detail between pathologists varies. Considering the algorithm train using a voxel size of 0.5 x 0.5 x 0.5 mm, it is not contributory to delineate more detailed than 0.5mm. To reduce variation in precision between pathologists the scale at which tumorous areas are annotated should be either **1 or 2 mm** (visible in the right down corner, as shown in the figure below)

Additionally, to prevent overdetailed annotation and reduce time-intensity, the thickness of the polygon line is set to 0.2mm.

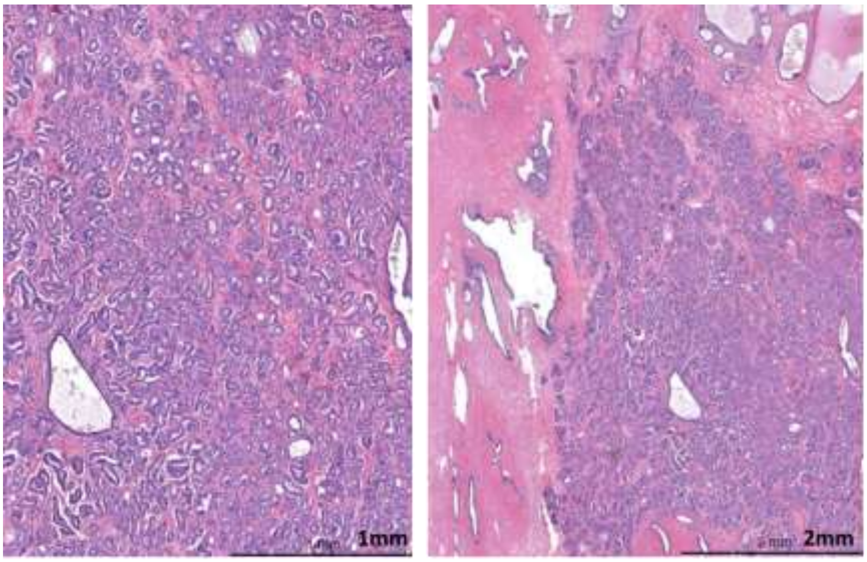

Cribriform growth vs. Intraductal carcinoma

Differentiating between cribriform growth and IDC is difficult if only HE-stained pathology slides are available. However, both growth patterns are highly clinically relevant. Therefore, the panel has decided to not differentiate between the two, but to annotate these areas as containing cribriform growth and/or IDC.

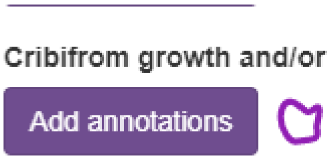

Supplement: Supplementary file 1 — (PDF 980 kb) [file 428_2023_3589_MOESM1_ESM.pdf]
